# Etiology of chronic urticaria: the Ecuadorian experience

**DOI:** 10.1186/s40413-017-0181-0

**Published:** 2018-01-03

**Authors:** I. Cherrez Ojeda, E. Vanegas, M. Felix, V. Mata, S. Cherrez, D. Simancas-Racines, L. Greiding, J. Cano, A. Cherrez, Juan Carlos Calderon

**Affiliations:** 1 Universidad Espíritu Santo, Km. 2.5 vía La Puntilla, Código postal: 0901-952 Samborondón, Ecuador; 2 Respiralab, Respiralab Research Group, Guayaquil, Ecuador; 3 School of Medicine, University of Heidelberg, Heidelberg, Germany; 4 Centro de Investigación en Salud Pública y Epidemiología Clínica. Facultad de Ciencias de la Salud Eugenio Espejo, Universidad Tecnológica Equinoccial, Quito, Ecuador; 5 Instituto Argentino de Alergia e Inmunología, Buenos Aires, Argentina; 6 University Hospital, Dermatology Department, Rostock, Germany

**Keywords:** Chronic urticaria, Chronic spontaneous urticaria, Chronic inducible urticaria, Autoimmune thyroid diseases, Urticarial vasculitis, Antihistamine

## Abstract

**Background:**

The purpose of this study was to identify chronic urticaria (CU) etiologies and treatment modalities in Ecuador. We propose that the sample distribution fits the expected one, and that there is an association between the etiology and its treatment.

**Methods:**

We performed a retrospective study involving 112 patients diagnosed with CU using a Checklist for a complete chronic urticaria medical history. Demographic and clinical variables were collected. The etiology of CU was classified using the EAACI/GA2LEN/EDF/WAO guideline. Descriptive analyses were performed for demographical and clinical variables. Chi square tests were applied to analyze the fit of distribution and the independence of variables. *P* values less than 0.05 were considered significant.

**Results:**

Among all the patients, 76.8% were diagnosed with chronic spontaneous urticaria (CSU), of which 22.3% had a known etiology or possible exacerbating condition. Food allergy was identified as the most common accompanying condition in patients with CSU (10.7%) (*p* < 0.01).. On the other hand, 23.2% inducible urticarias (CIndU) were indentified; dermographism was the most common (10.7%) (*p* < 0.01).

Regarding treatment regimens, sg-H1-antihistamines alone represented the highest proportion (44.6%). The combination of any H1-antihistamine plus other drug was a close second (42.0%) (*p* < 0.01). Almost 48% of CSUs of unknown etiology were treated with any antihistamine plus another drug. In patients with known etiology, sg-antihistamines alone (44.0%) was the most common management. In addition, 53.8% of CIndUs were treated with sg-antihistamines alone. Though, these associations were not statistically significant.

**Conclusion:**

CSU is the most frequent subtype of CU. Modern non-sedating antihistamines in licensed doses are the drug of choice. Nevertheless, a great proportion of patients require the addition of another type of medication.

## Background

Urticaria is defined by the presence of hives that appear and resolve within 24 h. Urticarial lesions can be circumscribed, raised, erythematous plaques, with central pallor. They can adopt different shapes and sizes (round, annular, or serpiginous), and are characterized by three main features: swelling and erythema; itching/burning sensation; spontaneous resolution within 24 h [1].

With regard to the duration of urticaria, it can be classified as “acute” (< 6 weeks) or “chronic” (> 6 weeks) [2]. Among patients with chronic urticaria, ≤ 40% can have accompanying episodes of angioedema (defined as a sudden swelling of the deep dermis in well-circumscribed areas like the lips, periorbital area, extremities, and genitals) [3, 4].

According to its underlying etiology, chronic urticaria is classified in two main groups: (i) chronic spontaneous urticaria (formerly known as “chronic idiopathic urticaria”), and (ii) inducible urticaria (including cold, delayed pressure, solar, heat, vibratory, cholinergic, contact, and aquagenic) [1].

Despite recent updates to the management guidelines for urticaria, it remains a challenge for healthcare providers to diagnose and identify each subtype of chronic urticaria due to the broad spectrum of clinical manifestations, and the possibility that several subtypes of the disease coexist in the same patient [5, 6]. Among physicians in Ecuador, a recent study suggested a low awareness of existing guidelines, resulting in poor knowledge of how to diagnose and treat the disease. It seems that the limited time per consultation (specially in public hospitals due to the volume of patients), together with low participation in medical meetings and conferences, led to poor adherence and application of current guidelines. Thus, patients were less likely to receive the recent evidence based treatments and diagnostic approaches [7].

With regard to treatment, two major research teams have published guidelines based on the available evidence and expert opinion [5, 6]. The US Joint Task Force on Practice Parameters (JTFPP) promotes a four-step approach, whereas guidelines set by EAACI/GA^2^LEN/EDF/WAO (European Academy of Allergology and Clinical Immunology, Global Allergy and Asthma European Network, European Dermatology Forum and World Allergy Organization) advocate a simplified three-step approach. Both guidelines agree on second-generation H1 antihistamines as the cornerstone and first-line therapy for chronic urticaria [4]. Treatment failure can prompt a dose increase of up to fourfold according to European guidelines or, in the case of the US guidelines: addition of another second-generation antihistamine, combination therapy with a first- and second-generation H1 antihistamine, or the addition of a leukotriene receptor antagonist as the next step. Both guidelines also agree upon the inclusion of omalizumab, cyclosporine, corticosteroids, and immunosuppressants to treatment if the initial regimen fails [8, 9]. However, in developing countries, where access to omalizumab is not provided by medical insurance, the affordability is very low [10, 11].

Data regarding the prevalence, demographics, and clinical characteristics of patients with chronic urticaria in Latin America, specifically in Ecuador, is limited. We aimed to fill this knowledge gap by describing the most common features and treatment choices of patients with chronic urticaria in Guayaquil, Ecuador.

## Methods

We carried out a retrospective study involving 112 patients diagnosed with chronic urticaria from 2005 to 2016 at Respiralab Research Center, Guayaquil-Ecuador. Demographic and clinical variables such as age, sex, years with the disease, type of urticarial, and medications were collected using medical records from the institution. The etiology of chronic urticaria was classified using EAACI/GA^2^LEN/EDF/WAO [5]. The diagnosis was effected using a checklist designed for chronic urticaria [12]. These check list items covered two main areas: Essential features for anamnesis and diagnosis of CU and typical symptoms/parameters or characteristics according to CU subtype, etiology, and laboratory findings. We developed an easy-to-use tool to support the early correct diagnosis and management of CU and facilitate healthcare providers/physicians’ diagnostic workup, clinical approach and allow to select the best approach for treatment in patients with CU.

Medications were subclassified into four groups: first-generation H1 antihistamine alone; second-generation H1 antihistamine alone; first- and second-generation H1 antihistamine combined; any H1 antihistamine with other types of medications (including, but not limited to, corticosteroids, topical agents, leukotriene inhibitors, and biologic therapy). These therapeutic modalities apply only to treatment initiation; no follow up or treatment modifications are described in this study.Descriptive analyses (frequency, percentage, standard deviation) were carried out for demographic and clinical variables. The chi-square test was applied to analyze the fit of distribution as well as the independence of variables. All statistical analyses were carried out using SPSS v24.0 (IBM, Armonk, NY, USA). *P* < 0.05 was considered significant.

## Results

Among the 112 patients studied, 69.6% were female and 30.4% were male, with the age ranging from 14 to 73 (mean, 35.8; SD, 13.8) years. The mean duration of suffering from chronic urticaria was 1.6 (SD 2.2) years (Table 1).

**Table 1:** – Demographic information of studied population

Characteristics	Patients (n=) n (%)
Age (years)	35.8 (13.8)
Years with urticaria	1.6 (2.2)
Gender	
Male	34 (30.4)
Female	78 (69.6)

### Urticaria type

In our data, 76.8% of patients were diagnosed with chronic spontaneous urticaria and 23.2% with chronic inducible urticaria (*p* = 0.001) (Table 2).

**Table 2:** – Urticaria diagnosis of studied population according to the EAACI/GA^2^LEN/EDF/WAO guidelines

Urticaria type	Overall patients (*n* = 112) n (%)
Chronic Spontaneous Urticaria	86 (76.8)
CSU of unknown etiology	61 (54.5)
CSU of known etiology	25 (22.3)
CSU with food allergy	12 (10.7)
Drug associated Urticaria	5 (4.5)
Thyroid Associated Urticaria	4 (3.6)
Chronic Autoimmune Urticaria	4 (3.6)
Chronic Inducible Urticaria	26 (23.2)
Symptomatic Dermographism	12 (10.7)
Cold Urticaria	2 (1.8)
Delayed pressure urticaria	2 (1.8)
Solar Urticaria	1 (0.9)
Vibratory Angioedema	6 (5.4)
Cholinergic Urticaria	3 (2.7)

All data are presented as frequencies and percentages. The chi-square goodness-of-fit test indicated that the proportions of urticaria subtypes diagnosed in the study were statistically significant (*p* = 0.00). *CSU* chronic spontaneous urticaria

Among all patients, 54.5% of cases had chronic spontaneous urticaria of unknown etiology, whereas the etiology was identified in 22.3%. With regard to the known etiology, food allergy was a relevant accompanying condition in 10.7% of all cases of chronic urticaria, while drugs accounted for 4.5% of cases. CSU with food allergy, described those patients presenting with chronic urticaria and accompanying food allergy, which was assessed through anamnesis and specific IgE [13]. Although the role of food allergy in chronic urticaria is highly controversial at the moment, current guidelines include immune mediated type I reactions (drugs, food, infections) in the diagnostic workup for CSU patients [5]. For this reason, we believe it is necessary to include food allergies as relevant conditions in our patients. Further research is needed to improve our understanding between food and determine their possible role, if any, in the pathogenesis of CSU.

**Fig. 1:**
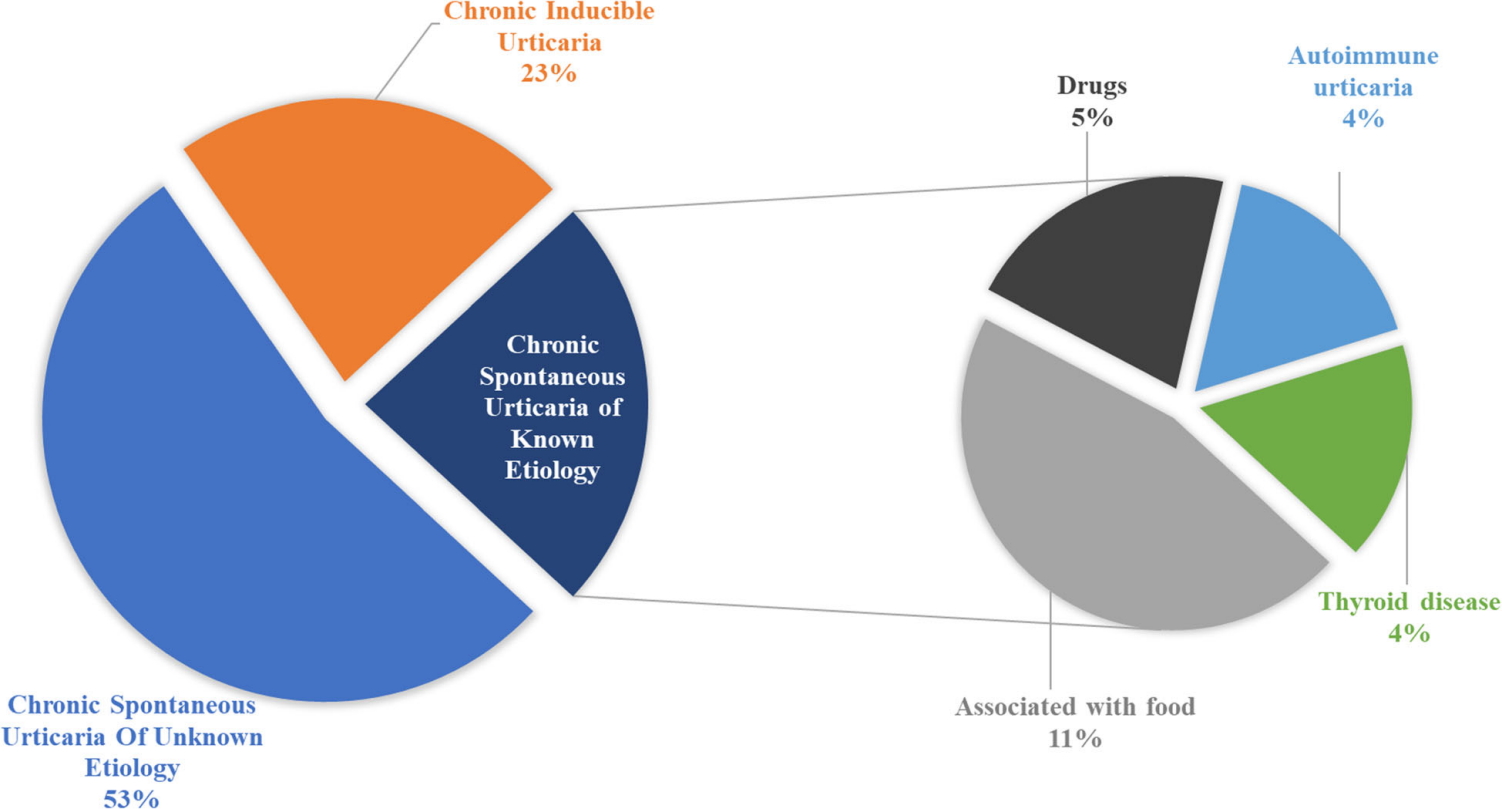
– Proportion of chronic urticaria etiologies in studied population

On the other hand, drug associated urticaria included patients with chronic urticaria triggered by drug allergy, which was diagnosed through careful anamnesis and confirmed by urticaria symptoms resolution after removal of the offending drug, as suggested by the EAACI/GA2LEN/EDF/WAO guideline [5]. Also, 3.6% of cases were associated with chronic autoimmune urticaria, while 3.6% were associated with thyroid disease (*p* = 0.00) (Fig. 1).

The most prevalent type of inducible urticaria was dermographism (10.7%), followed by vibratory angioedema (5.4%), cholinergic urticaria (2.7%), with 1.8% for both delayed-pressure urticaria and cold urticaria, and 0.9% for solar urticarial (*p* = 0.00) (Table 2).

### Treatment

With regard to treatment subgroups, the most prescribed regimen was a second-generation H1 antihistamine alone (44.6%). The combination of any H1 antihistamine (regardless of generation) plus another type of treatment (including autologous blood, topical agents, corticosteroids, anti-leukotriene agents, hydroxychloroquine or omalizumab) was also used widely (42.0%). A combination of a first- and second-generation H1 antihistamine was used in 10.7% of cases, whereas only 2.7% were prescribed with a first-generation antihistamine alone (*p* = 0.00) (Table 3).

**Table 3:** – Treatment modality prescribed in studied population

Treatment	Overall patients (n = 112) n (%)
Sg H1-antihisatmine alone	50 (44.6)
Sg H1-antihisatmine + another drug	47 (42.0)
Fg H1-antihistamine + Sg H1-antihisatmine	12 (10.7)
Fg H1-antihisatmine	3 (2.7)

All data are presented as frequencies and percentages. The chi-square goodness-of-fit test indicated that the proportions of treatment modalities prescribed in the study were statistically significant (p = 0.00). Fg H1-antihistamine, first-generation H1 antihistamine; Sg H1-antihisatmine, second-generation H1 antihistamine

### Urticaria type and treatment

**Fig. 2:**
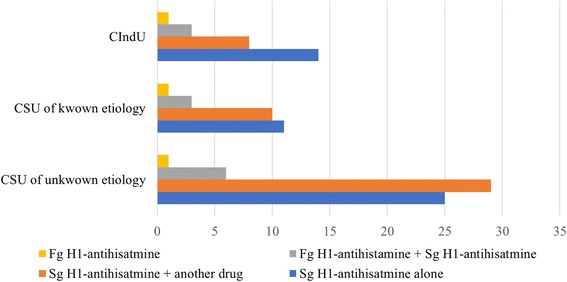
– Frequencies of treatment modalities prescribed by chronic urticaria subtype

Almost 48% of patients diagnosed with chronic spontaneous urticaria of unknown etiology were treated with any antihistamine plus another drug, and 41.0% were treated with a second-generation antihistamine alone. However, for patients with known etiology, the most common treatment was a second-generation antihistamine alone (44.0%). In addition, 53.8% of patients with chronic inducible urticaria were prescribed a second-generation antihistamine alone, and 30.8% required an antihistamine associated with another medication. However, this association between the type of urticaria and treatment was not statistically significant (*p* = 0.76) (Fig. 2).

## Discussion

Chronic urticaria is widely recognized as a relatively common disease that necessitates consultation with a dermatologist and/or allergist. Maurer and coworkers suggested that 66–93% of patients with chronic urticaria are diagnosed with the chronic spontaneous variant, 4–33% with physical urticaria, and 1–7% with cholinergic urticaria [14]. Our results are similar to the data obtained from the study by Maurer and colleagues.

Among chronic urticaria in Latin America, our demographic findings were comparable with the study by Gomez et al.; patients were were predominantly women, with a median age ranging in the 30s, and in average one and a half years with the disease (1.7 years in the Argentinian records) [15]. Treatment schemes were also similar, most patients were prescribed with second generation H1 antihistamines as a first line therapy.

In a previous study conducted from 2002 to 2004 in Ecuador, 161 patients diagnosed with chronic urticaria were classified according to guidelines set by the British Society for Allergy and Clinical Immunology [16]. Approximately 60% of those patients had a diagnosis that was compatible with chronic idiopathic urticaria [17]. Those findings, translated to the new EAACI/GA^2^LEN/EDF/WAO classification, would suggest that the proportion of patients with chronic spontaneous urticaria has not changed from 2005 to the present day in our local setting.

It is difficult to determine the role of food as an allergen in the induction of chronic urticarias for four main reasons. First, identification of a food as a trigger depends on medical history, which is often unreliable. Second, the results of elimination diets are considered contradictory. Third, measuring the allergen-specific immunoglobulin (Ig)E level for food has a poor positive predictive value. Finally, the results of double-blind placebo-controlled oral food challenge are difficult to interpret [18]. Similar to the prevalence reported by Kaeser et al., we found ≈10% of cases CSU with food allergy in our study. However, other studies in Asiatic populations have reported a prevalence of 1.1 and 2.8% [19, 20].

The second most common etiology reported by our patients with chronic spontaneous urticaria was induced by drugs. Kozel et al. published a study with 220 adults diagnosed with urticaria, of which 9.0% of cases were caused by drug allergies [21]. In the present study, the prevalence of drug-induced urticaria was more than double compared with that in our previous study (4.5% vs. 2%) [17]. Jares and colleagues reported that, in a Latin American population, urticaria and angioedema were the most prevalent clinical features (71%) in hypersensitivity reactions triggered by drugs. Nonsteroidal anti-inflammatory drugs and antibiotics were the most frequently used drugs in those patients. These drugs are sold over-the-counter, which can encourage their abuse [22].

Confino-Cohen et al. found a strong association between chronic urticaria and major autoimmune diseases. They also reported thyroid diseases to be the most common autoimmune diseases in patients with chronic urticaria [23]. To illustrate this correlation better, Kim and colleagues revealed that individuals with autoimmune thyroid diseases (AITD) were more likely to develop chronic spontaneous urticaria than a normal population (hazard ratio, 1.46; 95% confidence interval, 1.25–1.70). Those data demonstrated a significant association between AITD and chronic spontaneous urticaria [24]. Furthermore, autoimmune hypothyroidism and autoimmune hyperthyroidism have been said to be associated with chronic spontaneous urticaria, although the former appears to be far more common (9.8%) [23]. We found that 7.2% of cases with chronic spontaneous urticaria were associated with autoimmune disease, of which 3.6% were due to thyroid disease. In our patients, we confirmed the diagnosis of AITD with antithyroid antibodies above the reference range independently of the level of thyroid hormones. Compared with our previous study, the prevalence of chronic spontaneous urticaria associated with thyroid disease was reduced (3.6% vs. 6.0%) [17].

There is a subgroup of patients with chronic spontaneous urticaria who have autoantibodies against IgE or its receptor: FcεRI [25]. These autoantibodies act as activators of mast cells, leading to their degranulation and intracellular pathway signaling. This pathogenic condition is most commonly referred in clinical practice as “chronic autoimmune urticaria” [26]. The presence of these autoantibodies may be clinically important in a group of severely affected, treatment-resistant patients who might benefit from immunomodulatory agents [27]. A position paper proposed that the ‘gold standard’ for the diagnosis of autoimmune chronic spontaneous urticaria should be a combination of: (i) a positive in vitro biologic test to demonstrate the functionality of autoantibodies (basophil histamine release test or the expression of a marker of basophil activation such as cluster of differentiation (CD)63 or CD203c using flow cytometry), (ii) positive autologous serum skin test (ASST) to demonstrate the in vivo relevance of mast-cell degranulation and the increase in capillary permeability, and (iii) a positive immunologic assay for autoantibodies against FcεRIα receptors (western blotting or enzyme-linked immunosorbent assay) to demonstrate the specificity of autoantibodies [28].

Autoimmunity has been reported to be an etiologic factor in 40–60% of cases of chronic spontaneous urticaria [29]. In our patients, we confirmed the diagnosis with an ASST interpreted according to international guidelines [5]. Briefly, 0.05 mL of serum and 0.05 mL of plasma were injected intradermally. Histamine was used as a positive control and physiologic (0.9%) saline solution as a negative control. A minimum difference of 1.5 mm in wheal size between the positive control and negative control after 30 min was considered to be a positive test [30]. Even though the ASST has a low positive predictive value (≈55.1%), it represents the only screening tool available in daily clinical practice for most physicians due to its simplicity and cost-effectiveness [31]. However, a positive ASST in a patient with chronic urticaria can only suggest “autoreactivity” because its primary objective is to exclude the diagnosis [30]. Unfortunately, as in many other Latin American countries, we do not have confirmatory tests readily available to establish the actual diagnosis of autoimmune chronic spontaneous urticaria. In fact, only 12.9% of physicians in our country use the ASST, in line with guideline recommendations (7). This could explain why we diagnosed few patients with autoimmune chronic spontaneous urticaria.

Chronic inducible urticaria is characterized by itchy wheals, flare-type skin reactions, and/or angioedema induced by external physical factors. The latter can be mechanical (friction, pressure, vibration), thermal (cold, heat) stimuli, or electromagnetic radiation (solar radiation) [32]. Abajian and colleagues estimated the prevalence of chronic inducible urticaria to be 13.1–14.9% among patients with chronic urticaria [33]. Our findings are similar to those of Sanchez et al., who reported a prevalence of 36.3% among Latin American patients [34].

The most prevalent type of chronic inducible urticaria is symptomatic dermographism [35]. The latter was represented in 9.7% of our patients, but this prevalence was much lower than that reported by Sanchez et al., in which 24.8% tests tested positive for symptomatic dermographism [34]. Nevertheless, this prevalence was comparable with that reported in our previous study [17]. Environmental factors, such as geographic characteristics, could have had a key role in the differences between our results and the data of Sanchez et al. For instance, Bogotá in Colombia is located 2630 m above sea level and has an average temperature of 14.0 °C. Guayaquil is located 6 m above sea level and has an average temperature of 30.8 °C. Also, Schoepke et al. strongly suggested that dermographism might be precipitated by environmental factors or adverse events in life after observing that the peak age of onset of dermographism presented in the second and third decades of life [35].

In the study by Sanchez et al., cold urticaria was reported to be the second most prevalent type of chronic inducible urticaria. However, we found vibratory angioedema to be the second most prevalent type of chronic inducible urticaria. In addition, Sanchez hypothesized that exposure to cold environments might protect against cold urticaria. Interestingly, the prevalence of cold urticaria in our sample was one of the lowest, even though our patients were not exposed to low temperatures. Thus, we agree with Sanchez et al. that temperature or altitude are not the only determining factors in the development of chronic inducible urticaria.

Although urticarial vasculitis is not a subtype of chronic urticaria, it is a relevant syndrome to exclude in a patient whose chief complaint is an urticarial eruption accompanied by angioedema. In urticarial vasculitis, the lesions are painful, burning, and tender, with plaques lasting for > 24 h (sometimes ≤ 72 h). The wheals are associated with residual purpura or hyperpigmentation, and occasionally have a central dark-red or brown macule signifying underlying purpura and vasculitis. Other characteristics of urticarial vasculitis are swelling, residual bruising, and edema from focused pressure. Up to 81% of patients might present with extracutaneous symptoms, as reported in one retrospective study of 47 patients [36]. Skin biopsy must be undertaken to confirm or reject the diagnosis [37–39].

In our transversal study, we diagnosed 12 patients with urticarial vasculitis. If such patients were to be added to the sample of patients with chronic urticaria, it would represent 9.68% of all diagnoses. Hence, we consider it relevant to include urticarial vasculitis in the differential diagnosis of chronic urticaria. Thus, we highlight how such an entity must always be ruled out.

Despite the recent update of the EAACI/GA^2^LEN/EDF/WAO guidelines for chronic urticaria, the management of chronic spontaneous urticaria differs among various parts of the world [5, 7, 40]. As with clinical guidelines, published expert opinions recommend second-generation antihistamines as the preferred first-line treatment for chronic urticaria [41]. Patients who have unsatisfactory responses to standard doses of second-generation antihistamines should receive doses up to fourfold higher than the standard dose before alternative therapies are considered [5]. However, the addition of an H2-antagonist or a first-generation antihistamine to be taken at bedtime is another possibility [6].

In our study, a second-generation H1 antihistamine alone was the most common medication type prescribed, followed by antihistamines combined with other types of treatment (including autologous blood, topical agents, corticosteroids, antileukotriene agents, hydroxychloroquine or omalizumab). In a recent study, we found that only one-third of physicians reported using regular doses of second-generation H1 antihistamines as first-line treatment, and that specialists (dermatologists/allergologists) prescribed them more frequently [7]. Conversely, only 12.9% of physicians prescribed second-generation H1 antihistamines at higher doses when treating patients with chronic urticaria as second-line treatment [7].

Increasing the dose of second-generation H1 antihistamines up to fourfold is a relatively new recommendation. Nevertheless, physicians are not sufficiently confident to use this approach, in part because increasing the dose of antihistamines improves the control of pruritus significantly but does not reduce the number of wheals [42]. Moreover, the weaknesses of clinical studies and their significant heterogeneity limits the consistency of findings to support this approach [42]. The fear of possible side effects using higher doses of second-generation H1 antihistamines is another important factor among physicians [42].

The prevalence of combined use of first- and second-generation H1 antihistamines, or the use of a first-generation H1 antihistamine alone, were relatively low for our patients (10.7, and 2.7%, respectively). In Ecuador, some physicians (particularly pediatricians) commonly use first-generation H1 antihistamines to treat chronic urticaria [7]. Also, some practice guidelines continue to recommend them [6]. Ferrer and colleagues noted that hydroxyzine was the second most frequently prescribed drug, with no difference in the prevalence of prescribing between dermatologists and allergists [43]. A possible explanation could be that first-generation H1 antihistamines cost less than second-generation H1 antihistamines in Ecuador.

Chronic inducible urticaria appears to be more resistant to standard doses of antihistamines compared with chronic spontaneous urticaria, thus necessitating higher doses to achieve symptom control [44]. In our patients, antihistamines alone achieved control of the symptoms of chronic inducible urticaria whereas, for chronic spontaneous urticaria, a combination of different medications was needed. Unfortunately, we cannot draw any conclusions from this observation.

During the last 14 years, 273 patients have been diagnosed with chronic urticaria at our center. No significant changes in the etiology of urticaria have been found. Even though urticarial vasculitis is not a type of urticaria, it was identified as the principal differential diagnosis in our cross sectional study.

Our has study has some limitations. First, we didn’t assess severity of the disease using the Urticaria Activity Score (UAS) before and after treatment. By the time these patients were diagnosed and treated, an official Spanish version of UAS wasn’t available. Instead, we used a Visual Analog Scale (VAS) to assess the pruritus and wheals severity. Second, for clinical entities such as food and drug associated urticaria, we performed the diagnosis mostly through anamnesis. A careful clinical history is recommended for both etiologies, but it relies on the patient’s awareness and memory, which is a major downside of major concern for drug associated urticaria. For food allergy, we complemented diagnosis with specific IgE. Even though oral food challenge (OFC) is the gold standard for food allergy diagnosis, we didn’t perform such test. For drug associated urticaria, we interpreted symptoms resolution after offending drug removal as a confirmatory finding for diagnosis, though, more objective tests such as Prick test or in vitro testing could have been helpful. Third, we didn’t provide any data concerning follow up, severity, response or quality of life. This study focusses mainly on the distribution of urticaria etiology and first therapeutic regimen prescribed.

## Conclusions

Chronic spontaneous urticaria is one of the most prevalent subtypes of chronic urticaria. Non-sedating antihistamines in licensed doses are the drug of choice. Nevertheless, a considerable proportion of patients require the addition of another type of medication.

There is a need to elucidate the other factors that contribute to the development of chronic urticaria and the the optimal management of this disease. Furthermore, we believe that urticarial vasculitis must be excluded as a differential diagnosis of any subtype of chronic urticaria.

## Abbreviations

AITD: Autoimmune thyroid diseases
ASST: Autologous serum skin test
CINDU: Chronic Inducible urticaria
CSU: Chronic spontaneous urticaria
CU: Chronic Urticaria
EAACI/GA^2^LEN/EDF/WAO: European Academy of Allergology and Clinical Immunology, Global Allergy and Asthma European Network, European Dermatology Forum and World Allergy Organization
JTFPP: Joint Task Force on Practice Parameters
SD: Standard deviation
